# Antimicrobial and Antibiofilm Effects of Silver-Copper Nanoparticles Obtained by Green Synthesis Against Streptococcus mutans

**DOI:** 10.7759/cureus.58368

**Published:** 2024-04-16

**Authors:** Büşra Merve Sarıtaş, Aybek Yiğit, Ayşe Karacalı Tunç, Fetiye Kolaylı

**Affiliations:** 1 Medical Microbiology, Kocaeli University, Kocaeli, TUR; 2 Research Laboratory Application and Research Center (ALUM), Iğdır University, Iğdır, TUR; 3 Faculty of Dentistry, Iğdır University, Iğdır, TUR

**Keywords:** silver nanoparticles, dental caries, antibiofilm assay, antimicrobial effect, copper nanoparticles

## Abstract

Introduction*: *Oral diseases affect billions worldwide, with dental caries being a significant concern. Silver-copper nanoparticles (Ag-Cu NPs) synthesized from pitaya plant extract offer a potential solution due to their antimicrobial properties. This study aimed to evaluate the inhibitory effects of Ag-Cu NPs on *Streptococcus mutans *(*S. mutans*), a major contributor to dental caries.

Methodology: Ag-Cu NPs were synthesized using a green chemical method and characterized using a scanning electron microscope (SEM), ultraviolet-visible (U-vis) spectrophotometer, and Fourier Transform Infrared Spectroscopy (FT-IR). The minimum inhibitory concentration (MIC) against *S. mutans* was determined using the broth microdilution method, while the antibiofilm effect was assessed by the crystal violet method.

Results: The synthesized Ag-Cu NPs demonstrated antimicrobial activity with an MIC of 128 µg/ml, significantly inhibiting *S. mutans* biofilm formation by up to 94% at a concentration of 256 µg/ml. Characterization studies confirmed the successful synthesis of Ag-Cu NPs with spherical morphology.

Conclusions: These findings highlight the potential of Ag-Cu NPs as a novel approach for combating dental caries by targeting* S. mutans* biofilms. Further research is warranted to explore their safety and efficacy in clinical settings.

## Introduction

According to the World Health Organization's Global Oral Health Status Report for the year 2022, oral diseases have a significant impact on approximately 3.5 billion people worldwide, with approximately 75% of those affected residing in middle-income countries both in developed and economically disadvantaged countries, dental caries remain a significant public health concern [1]. Following the identification of *Streptococcus mutans* (*S. mutans*) as the causative agent of dental caries, considerable attention has been directed towards targeting this bacterium in disease prevention efforts through the use of antimicrobial agents and vaccine development [2]. In conclusion, there is a need for a substance that does not promote bacterial resistance and exhibits strong bactericidal properties. Nanoparticles (NPs) are strong candidates for such substances. NPs with sizes ranging from 1 to 100 nanometres (nm) can be synthesized by different methods. It is known that when performing the synthesis process with a green chemical method, fewer chemicals are used and are less harmful to the environment [3,4]. *S. mutans*, which causes tooth decay, is an acidogenic bacterium that forms biofilms. One of the most notable attributes of *S. mutans* is its ability to form biofilms. Biofilms are complex communities of microorganisms encased in a self-produced extracellular matrix that adhere to both natural teeth and dental restorations [5]. This biofilm matrix, composed of polysaccharides, proteins, and extracellular DNA, not only acts as a protective shield against various environmental challenges, such as fluctuations in pH and nutrient availability but also enhances the adhesive properties of bacteria. This allows *S. mutans* to firmly adhere to dental surfaces, facilitating its colonization and further contributing to the acidogenic and aciduric environment within the biofilm [5,6].

In this study, we used Ag-Cu NPs synthesized from pitaya (Hylocereus spp.) plant extract to assess their inhibitory effects on* S. mutans*. We used a standard strain of *S. mutans* to evaluate the bactericidal activity and antibiofilm effect of these NPs. To our knowledge, there is not much research on the bactericidal activity and antibiofilm activity of Ag-Cu NPs against *S. mutans.*

## Materials and methods

Preparation of plant extracts

The leaves of the pitaya plant were collected by cutting and then thoroughly washed with distilled water. Afterwards, they were stored in an oven at a low temperature (35°C) for five days. The collected leaves were finely blended. Subsequently, the blended leaves were placed in 250 ml Erlenmeyer flasks containing 100 ml of distilled water and kept at 70°C for 30 minutes with constant stirring using a magnetic stirrer, while the flasks were covered with aluminum foil. After 30 minutes, the plant sample in the Erlenmeyer flasks was subjected to filtration using filter paper with a pore size of 0.22 mm. The filtrate obtained after filtration was stored at +4°C for later use [7].

Synthesis of Ag-Cu NPs

After the extraction process, 10 ml of the plant filtrate was removed, followed by 90 ml of distilled water, 20 mg of silver nitrate, and 20 mg of copper (II) chloride. These components were mixed using a magnetic stirrer under room conditions and covered with aluminum foil for 12 hours. Upon the addition of silver nitrate, the color of the copper (II) chloride solution changed from light blue to dark blue, and after the addition of the extract, it turned dark green. After 12 hours, the sample was centrifuged at 4,000 rpm for five minutes three times (twice with water and once with ethyl alcohol). The obtained solid material was then dried in an oven at 60°C for characterization [7-10]. 

Characterization of Ag-Cu NPs

Scanning electron microscope: The structural characterization of the samples was carried out using a scanning electron microscope (SEM) (Carl Zeiss Sigma 300, Germany) with an in-lens detector at an accelerating voltage of 10 kV.

Ultraviolet-visible spectroscopy: Optical properties and absorption spectra of the produced Ag-Cu NPs were determined. Characterization was performed using an ultraviolet-visible (UV-vis) spectrometer (Cary 60 Agilent Technologies, USA) with wavelengths ranging from 300 to 800 nm.

Fourier Transform Infrared Spectroscopy: In this characterization step, the model Fourier Transform Infrared Spectroscopy (FT-IR) (Agilent Cary 630 Agilent Technologies, USA) was used.

Antimicrobial activity

The minimum inhibitory concentration (MIC) of the NPs was determined in 96-well U-bottomed microplates using the broth microdilution method. 100 µl of brain heart infusion broth (BHIB) were added to each well. A twofold serial dilution was prepared by adding 100 µl of Ag-Cu bimetallic NPs at a concentration of 1024 µg/ml to the wells. A negative control was prepared without the addition of NPs. Chlorhexidine (CHX) (0.02%) was used as a positive control. Additionally, for comparison purposes, a watery solution of pitaya extract was used. The *S. mutans *HF 676 strain was used in the study. The bacterium, taken from the -80°C stock, underwent two subcultures. For the liquid microdilution method, a bacterial suspension was prepared in 0.9% NaCl solution from growing colonies, achieving turbidity of 0.5 McFarland, and was diluted at a 1:10 ratio. In addition to the last well, 5 µl of the diluted bacterial suspension was added to all the wells. The microplates were incubated at 37°C in a candle jar (5% CO_2_) for 24 hours. The lowest concentration with no visible growth was determined as the MIC.

Antibiofilm effect of Ag-Cu NPs by the crystal violet method

The effect of NPs on biofilm formation was evaluated in 96-well flat-bottomed microplates. Then, 180 µl of BHIB (2% sucrose) was added to all the wells. To determine the inhibitory effect on biofilm formation, NP solutions were prepared at a concentration of 256 µg/ml and then diluted twofold before being added to the wells. A 20 µl bacterial suspension was inoculated, followed by incubation. After incubation, planktonic bacteria were removed from the medium. The wells were washed three times with phosphate-buffered saline (PBS). After drying the microplates, 200 µl of 95% methyl alcohol was added. Crystal violet (0.1%) was added to the wells for 15 minutes of staining. Following the staining process, the wells were washed three times with distilled water, and 200 µl of ethyl alcohol was added. After allowing the plates to stand, optical density (OD) values at 630 nm were measured using a multiwell spectrophotometer. Biofilm inhibition after treatment with NPs was quantitatively assessed using the following formula:

$\%\text{ Biofilm inhibition} = \left[ 1 - \left( \frac{\text{OD} \text{ of cells treated with NPs}}{\text{OD} \text{ of the negative control}} \right) \times 100 \right]$

## Results

Characterization of Ag-Cu NPs

**Figure 1:**
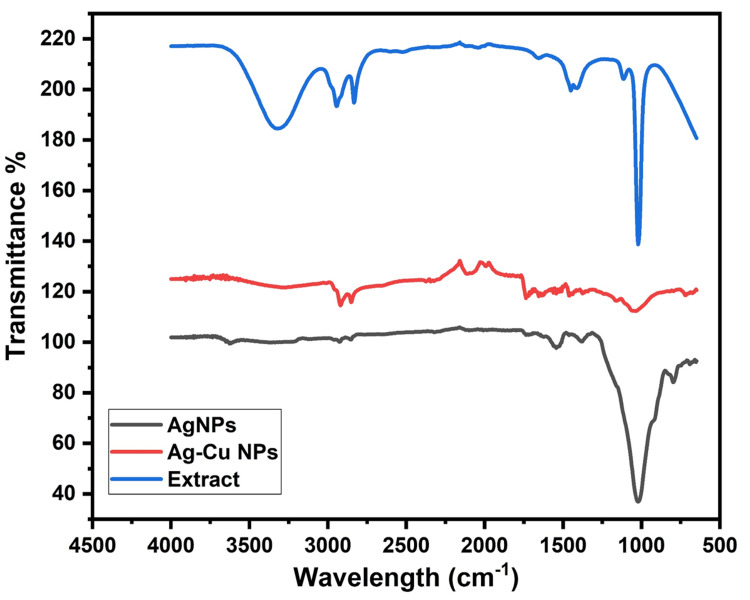
FT-IR image of pitaya extract and Ag-Cu NPs, Ag NPs FT-IR: Fourier Transform Infrared Spectroscopy; NP: Nanoparticle

FT-IR: A model FT-IR device (Agilent Cary 630 Agilent Technologies, USA) and FT-IR devices of the PerkinElmer Spectrum Two (KBr) series were employed in the characterization of the samples. The FT-IR spectrum of Ag-Cu NPs shows several bands characteristic of the stretching and bending vibrations of O-H, C-C, and C-O functional groups (Figure 1). According to the images obtained from the FT-IR device, strong vibrations between 3,000 and 4,000 cm^-1^ are associated with (OH) groups, strong vibrations between 1,500 and 2,000 cm^-1^ are associated with carbonyl groups of the acid group, and strong vibrations between approximately 1,500 and 2,000 cm^-1^ are associated with amide groups. The peak at approximately 1,000 cm^-1^ is related to Ag-Cu NPs [11-15]. There is also slightly more flatness in the Ag-Cu FT-IR images.

**Figure 2:**
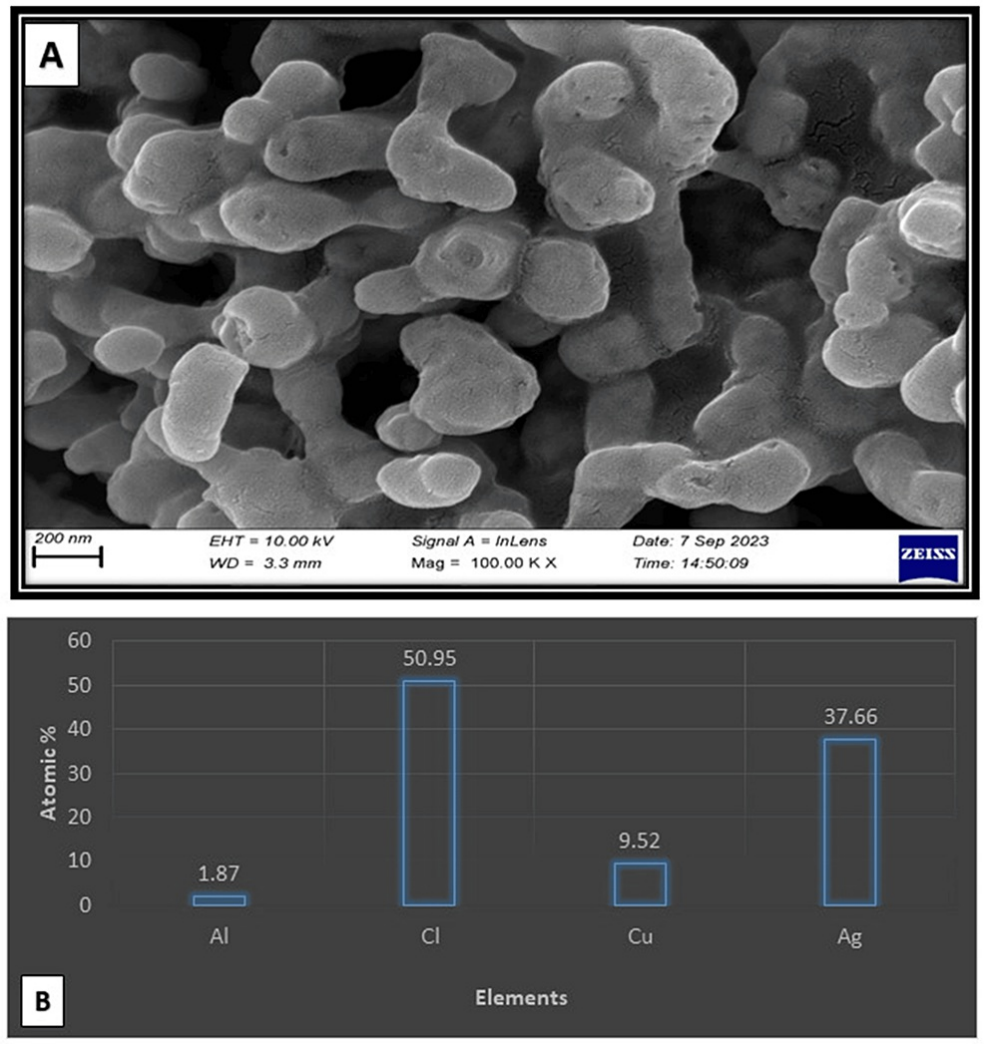
Images of Ag-Cu NPs produced by green synthesis using SEM (A) and SEM-EDX (B) NP: Nanoparticle; SEM: Scanning electron microscope

SEM: A Carl Zeiss Sigma 300 model SEM device was used to obtain morphological images. During imaging, spherical structures of the Ag-Cu NPs were observed. Additionally, the weight percentages of Ag and Cu were also determined from the SEM‒EDX images (Figure 2) [14]. According to SEM-EDX, the weight percentage of Ag was greater than that of Cu.

UV-vis: Spectroscopic studies were carried out with a model device UV-vis spectrophotometer (Cary 60 Agilent Technologies, USA). The peak obtained is similar to the peak obtained in the literature. In the spectrum obtained from the UV-vis device, the highest peak was attributed to Ag-Cu NP particles (Figure 4) [14].

**Figure 3:**
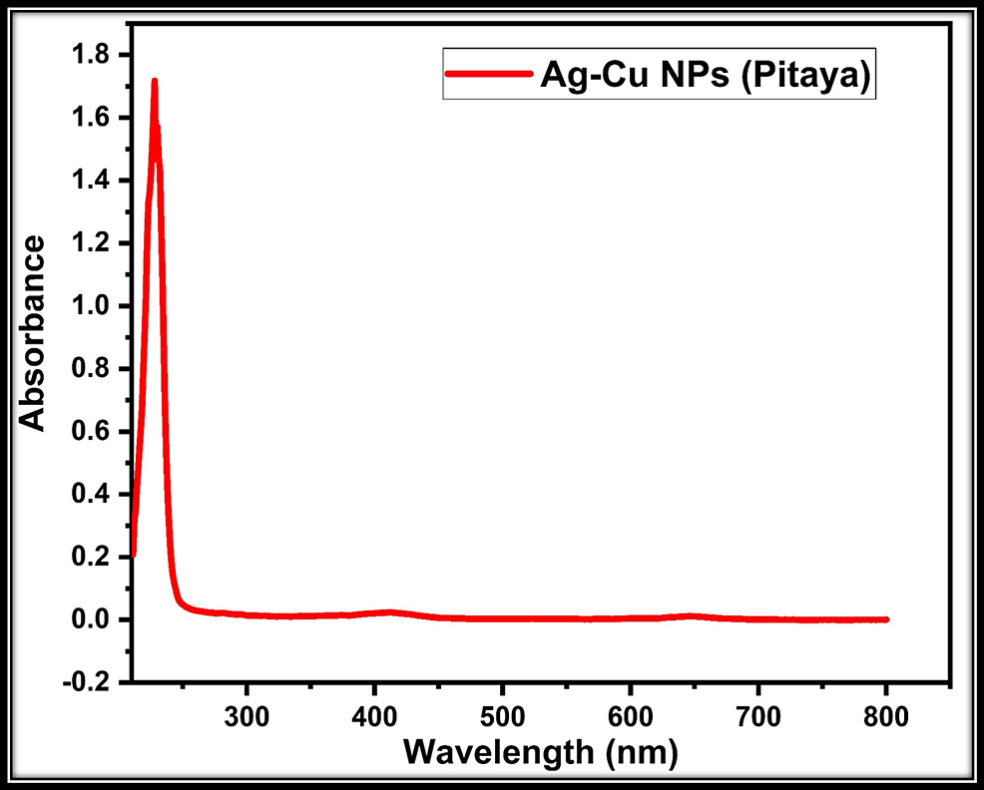
The images of the Ag-Cu NPs in the UV-vis spectrophotometer NP: Nanoparticle; UV-vis: Ultraviolet-visible

Antimicrobial activity of Ag-Cu NPs

It was tested that bacterial growth conditions were met by adding only bacteria to the positive control. Sterility control was also carried out to check that there was no contamination in the medium. No bacteria were added to the sterility control. The first well at which visible growth disappeared was determined as the MIC value. The MIC of Ag-Cu bimetallic NPs was 128 µg/ml. Pitaya extract did not exhibit any antimicrobial effects. CHX MIC is 0.0006%. It has been observed that bimetallic NPs have antimicrobial activity (Figure 3).

**Figure 4:**
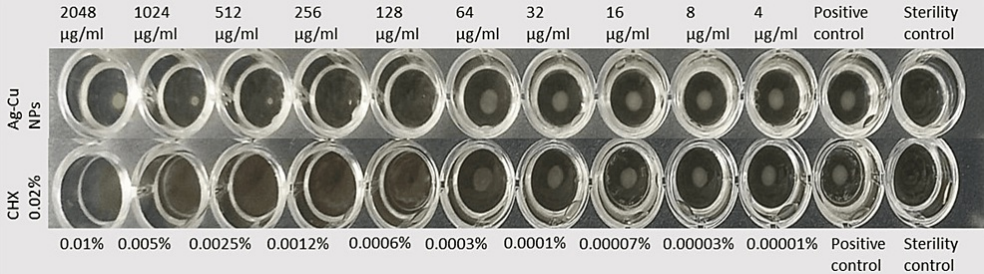
Antimicrobial activity of Ag-Cu NPs NP: Nanoparticle

The antibiofilm effect of Ag-Cu NPs

**Figure 5:**
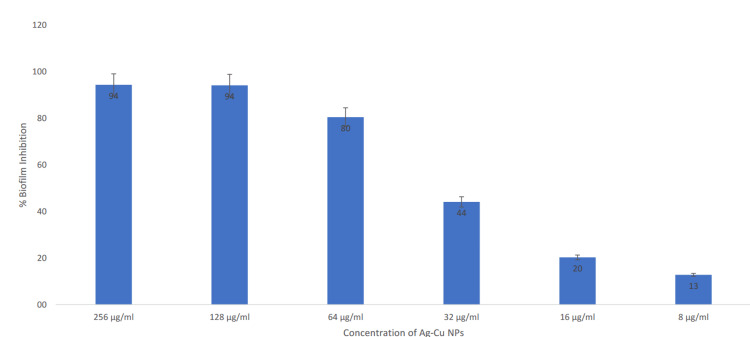
% biofilm inhibition of Ag-Cu NPs applied at different concentrations compared to the positive control NP: Nanoparticle

The results demonstrate the remarkable efficacy of synthesized Ag-Cu NPs in inhibiting biofilm formation by *S. mutans*. At a concentration of 256 µg/ml, the Ag-Cu bimetallic NPs exhibited a remarkable biofilm inhibition rate of 94%. Even at a lower concentration of 32 µg/ml, significant biofilm inhibition of 44% was observed (Figure 5).

## Discussion

Traditional methods for combating *S. mutans*, such as the use of antibiotics, have limitations, including the potential for resistance development. Ag-Cu NPs offer a new approach that does not promote bacterial resistance and are effective against *S. mutans*, the primary cause of dental caries, as highlighted in this study.

Biofilm formation by *S. mutans* is an important factor in the development of dental caries. These biofilms provide a protective shield for *S. mutans*, allowing it to thrive in the acidic microenvironment created by its lactic acid production [5]. The results of this study reveal the potent antibiofilm effects of Ag-Cu NPs, which inhibit the formation of these microbial communities. By eradicating biofilm development, Ag-Cu NPs can impede the colonization and growth of *S. mutans* on dental surfaces. This is a pivotal step in preventing dental caries, as biofilm disruption not only hampers the acidogenic environment but also increases the susceptibility of the bacterium to antimicrobial agents.

In a study conducted in 2023, the antibacterial and antibiofilm effects of silver nitrate, silver diamine fluoride, and Ag NPs on *S. mutans* were investigated, and the antimicrobial and antibiofilm activities of Ag NPs were found to be greater than those of other agents [16]. In our study, Ag-Cu NPs were found to have antimicrobial and antibiofilm effects. A higher antibiofilm effect was observed at higher concentrations. Investigated the antibiofilm and antibacterial activity of dissolved silver obtained from silver-coated titanium implants against *S. mutans* and reported that the amount of silver transferred to BHIB had an antimicrobial effect, while the antibiofilm activity was not significant [17]. Extracts of *Streblus asper, Cymbopogon citratus, Syzygium aromaticum*, and a mixture of the three herbs demonstrated antibacterial activity against *S. mutans* isolates by reducing bacterial biofilm formation and decreasing bacterial cell surface hydrophobicity. The formulated Ag NPs from the ethanolic extracts could enhance the antibacterial activities of the plant extracts [18]. Although no effect of pitaya extract was observed in our study, the antimicrobial and antibiofilm activity of Ag-Cu NPs on this plant was obtained.

In a study on the inhibition of bacteria that cause periodontitis, gingival patch mucoadhesion containing red dragon fruit peel extract (GP-RDFPE) inhibited *Porphyromonas gingivalis, Fusobacterium nucleatum*, and *Aggregatibacter actinomycetemcomitans *[19]*.* The inhibitory effects on *Porphyromonas gingivalis, Fusobacterium nucleatum,* and *Aggregatibacter actinomycetemcomitans* were significant at an RDFPE concentration of 6.25%, not at an RDFPE concentration of 3.125%. RDFPE at concentrations of 3.125% and 6.25% inhibited *Porphyromonas gingivalis, Fusobacterium nucleatum, and Aggregatibacter actinomycetemcomitans*, respectively. An RDFPE concentration of 6.25% is considered the MIC, and an RDFPE concentration of 12.5% is the MBC [19]. Topical cream obtained from Hylocereus polyrhizus extract has been reported to be effective in wound healing [20]. Hylocereus species are frequently used for different therapeutic purposes.

In the study by Espinosa-Cristóbal et al., Ag NPs exhibited greater antimicrobial effects on *S. mutans* at lower concentrations than gold or zinc [2]. In a study by Valodkar et al., Ag-Cu NPs showed antimicrobial effects on gram-positive and gram-negative bacteria [21]. The MIC of Strobilanthes crispus (SC)-mediated Ag NPs (SC-Ag NPs) from *S. mutans* has been reported to be 2.5 mg/ml [22].

Many studies support the fact that Ag NPs obtained via green synthesis are effective against *S. mutans*. Research conducted with two groups of NPs and NPs obtained from pitaya plants is limited. Although the observed antibiofilm and antimicrobial effects of Ag-Cu NPs hold promise in combating dental caries by targeting *S. mutans* biofilms, several key limitations warrant further investigation. Specifically, potential cytotoxicity concerns at higher NP concentrations and their long-term impact on the oral microbiota remain to be fully understood. These aspects necessitate comprehensive toxicity studies to ensure the safety of Ag-Cu NPs for oral use. Moreover, while in vitro studies provide valuable insights, translating these findings into clinical applications requires robust validation through in vivo models and subsequent clinical trials. These endeavors are essential to ascertain the safety, efficacy, and practical feasibility of incorporating Ag-Cu NPs into real-world oral health interventions.

## Conclusions

In our study, we evaluated NPs exhibiting antimicrobial and antibiofilm effects against bacteria, which are significant contributors to dental caries. However, further investigations should include cytotoxicity studies, particularly at higher concentrations of NPs, to ensure their safety for oral use. The synthesized NPs hold potential for integration into mouthwash pastes and filling materials to effectively prevent tooth decay. Notably, the utilization of green chemistry in NP synthesis represents a more environmentally friendly approach. The prevention of biofilm formation presents a promising strategy in addressing challenges associated with antimicrobial resistance and biofilm eradication in dental care. Future research endeavors could explore the drug candidacy of these NPs through comprehensive cytotoxicity assessments and studies employing experimental animal models. Such advancements will contribute significantly to the development of innovative approaches in dental caries prevention and treatment.

Additionally, studies involving in vivo models and clinical trials are necessary to confirm the safety and efficacy of these NPs in real-world oral health settings.
